# Free Radical Polymerization of Styrene and Maleimide Derivatives: Molecular Weight Control and Application as a Heat Resistance Agent

**DOI:** 10.3390/molecules30091863

**Published:** 2025-04-22

**Authors:** Jiawei Ding, Changlei Yang, Liqiong Zhou, Wenjing Li, Jiaqi Li, Cixiang He, Yufei Liu, Min He, Shuhao Qin, Jie Yu

**Affiliations:** 1 Department of Polymer Material and Engineering, College of Materials and Metallurgy, Guizhou University, Guiyang 550025, China; j2940261288@163.com (J.D.); 13385118086@163.com (C.Y.); 18586606304@163.com (L.Z.); m19198280547@163.com (W.L.); 15737871959@163.com (J.L.); 18485430132@163.com (C.H.); pec.shqin@gzu.edu.cn (S.Q.); pec.jyu@gzu.edu.cn (J.Y.); 2 National Engineering Research Center for Compounding and Modification of Polymeric Materials, Guiyang 550014, China

**Keywords:** P(N-phenylmaleimide-alt-styrene), heat-resistant agent, molecular weight

## Abstract

Poly (styrene-maleic anhydride) copolymers, due to their unique structure, are extensively functionalized and modified for preparing heat stabilizers, compatibilizers, and other functional additives. Using 4-methylpent-1-ene-2,4-diyl diphenyl (α-MSD) as a chain transfer agent, a series of molecular-weight-controlled maleic anhydride-derived styrene copolymers, poly(N-p-fluorophenylmaleimide-alt-styrene) (PFS) and poly(N-p-carboxylphenylmaleimide-alt-styrene) (PCS), were synthesized via free radical copolymerization. The molecular weights of PFS and PCS were adjusted to explore their impact on the properties of PFS/PA6 and PCS/PA6 blends. Gel permeation chromatography (GPC) analysis confirmed that α-MSD effectively regulated the molecular weights of PFS and PCS. PFS and PCS with lower molecular weights exhibited significantly reduced viscosity, with minimal impact on their thermal and mechanical properties.

## 1. Introduction

Radical polymerization proceeds via radical chain propagation; however, this method suffers from several limitations, including poor control over molecular weight, broad molecular weight distribution, and difficulty in accessing highly functionalized polymers [1,2,3,4]. Achieving precise molecular weight control in free radical polymerization (FRP) is critical for tailoring the properties of polymers, particularly for applications demanding high performance and durability [5,6,7,8]. Molecular weight regulation exerts significant influence on the overall performance of polymers, including thermal stability, mechanical properties, crystallization behavior, and electrical conductivity, among others [9,10]. Research findings indicate that molecular weight regulation in free radical polymerization can be achieved through the optimization of initiators, chain transfer agents, reaction conditions, and polymerization processes, among others [11,12,13]. Over the past two decades, controlled/living radical polymerization (CRP) has emerged as a powerful method for synthesizing polymers with well-defined structures, architectures, and narrow molecular weight distributions. Among the primary CRP techniques are nitroxide-mediated polymerization (NMP), atom transfer radical polymerization (ATRP), and reversible addition-fragmentation chain transfer (RAFT) polymerization [14,15,16,17,18,19]. Owing to its ability to precisely control the molecular weight, molecular weight distribution, and molecular structure of polymers by design, CRP has garnered significant attention and research interest [20,21]. While CRP offers an effective approach for synthesizing polymers with specific molecular structures, molecular weights, and narrow molecular weight distributions; this method is also accompanied by limitations, including more complex polymerization systems, more stringent polymerization conditions, higher costs, and a relatively limited scope of application [22,23].

This study examined a simple and effective method for regulating the molecular weight of styrene and maleimide derivative copolymers. The method involved the use of (4-methylpent-1-ene-2,4-diyl)dibenzene (α-MSD) as a chain transfer agent (CTA) in free radical polymerization. This approach allows precise control over the molecular weight and distribution of the resulting copolymers. Bhaswati and Basab synthesized highly pure 4-methylpent-1-ene-2,4-diyl) diphenyl (α-MSD) through a liquid–liquid biphasic reaction of α-methylstyrene (AMS) with acid catalysis [24,25]. Zhang Peng-Rong and Hideki Kurokawa et al. prepared α-MSD with high yield and selectivity by changing different acidic solvents and catalysts [26,27]. Yasumasa et al. investigated the addition-fragmentation chain transfer mechanism of α-MSD in styrene free radical polymerization, which showed that a polymer radical first adds to the terminal double bond of α-MSD, generating an addition radical [28]. Subsequently, the radical breaks apart, forming a polymer chain containing terminal double bonds and an isopropylbenzene radical. α-MSD was employed as an effective chain transfer agent in the free radical polymerization of polystyrene, a styrene–maleimide copolymer, and an acrylic acid-based copolymer to precisely control the molecular weight [29,30].

Malaysianimide, as a deficit-electron-affinitive diene monomer, readily reacts with various electron-rich vinyl monomers [31]. Styrene and maleimide derivative copolymers are of significant importance in high-performance materials due to their unique physicochemical properties. These properties are primarily attributed to the conjugated double bonds, aromatic rings, and highly polar carbonyl groups in their molecular structures. Owing to their superior characteristics, styrene and maleimide derivative copolymers are widely utilized in engineering applications such as photoresists, nanoparticles, polymer microspheres, and coatings [32,33,34,35]. Owing to their excellent thermal stability and inherent rigidity, styrene and maleimide derivative copolymers can enhance the thermal and mechanical performance of polymers through the formation of intermolecular forces when blended [36,37,38,39,40,41,42,43,44,45,46,47,48,49]. Polyamide 6 (PA6) and its composite materials, attributed to their excellent mechanical properties and wear resistance, have been extensively employed across various engineering applications. Nevertheless, under conditions involving elevated temperatures and exposure to intense mechanical forces, the thermal stability of PA6 remains a critical area necessitating further enhancement [40,41,42].

In this study, styrene and maleimide derivative copolymers were used as thermal stabilizers and blended with PA6 to enhance the thermal stability of PA6-based materials, thereby expanding the application scope of PA6. Additionally, chain transfer agents (CTAs) were incorporated during the polymerization of the styrene and maleimide derivative copolymers to regulate their molecular weight. This study investigated the effects of the molecular weight of the styrene and maleimide derivative copolymers on the properties of PA6 composites.

## 2. Results and Discussion

### 2.1. Synthesis of PFS and PCS Copolymers and Their Molecular Weight Control

**Scheme 1:**
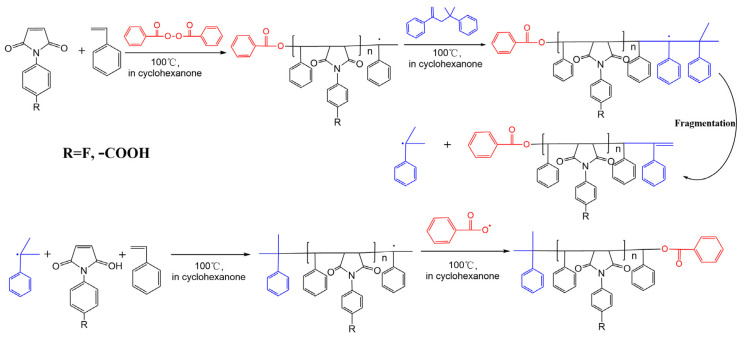
Copolymerization of PFS (or PCS) via solution polymerization in cyclohexanone at 100 °C.

Scheme 1 outlines the copolymerization of styrene with 4-FPMI and 4-CPMI via free radical polymerization copolymerization.

In principle, (4-methylpent-1-ene-2, 4-diyl) dibenzene (α-MSD) is a suitable chain transfer agent for the homopolymerization of styrene [43]. However, it was used as the chain transfer agent for the copolymerization of styrene with 4-FPMI and 4-CPMI in the present study. In Scheme 1, the chain transfer mechanism of α-MSD in PFS and PCS polymerization is illustrated. A polymer chain radical is generated by the reaction of maleic anhydride derivatives and styrene, initiated by BPO. The polymer chain radical then reacts with the susceptible double bond at the end of α-MSD to form an intermediate radical [44]. The intermediate radical decomposes to generate an isopropylbenzene radical and a polymer dead chain with carbon–carbon double bonds at the chain end. The isopropylbenzene radical further initiates the reaction of maleic anhydride derivatives and styrene to form a polymer chain radical, which finally couples with the decomposition radical of BPO to terminate the polymer chain.

**Figure 1:**
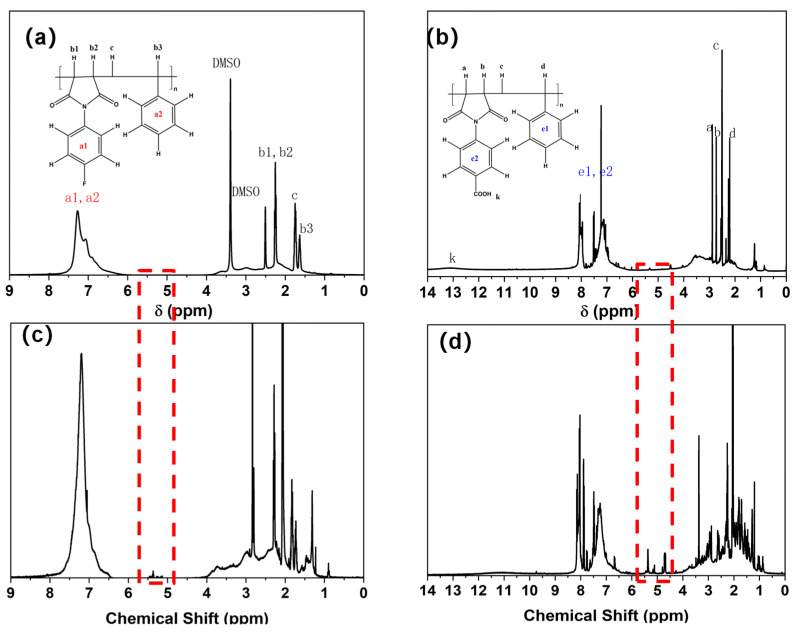
^1^H NMR spectra recorded for (**a**) PFS in DMSO and (**b**) PCS in CCl_3_D. (**c**) PFS/α-MSD in DMSO and (**d**) PCS/α-MSD in CCl_3_D.

Figure 1 records ^1^H NMR spectra of (a) PFS in DMSO and (b) PCS in CCl3D. In Figure 1a, 1.6 ppm to 1.8 ppm is the signal for protons on methylene and methylides attached to the benzene ring. Furthermore, the signals ranging from 6.5 ppm to 7.5 ppm can be attributed to the protons present on the benzene ring. The proton signal at 2.3 ppm can be assigned to the –CH–CH– group in the maleimide component. The structural elucidation of PFS can be derived from the aforementioned ^1^H NMR spectra [45,46,47]. The ^1^H NMR spectrum below Figure 1a exhibits signal peaks corresponding to the PFS structure, but there is also a signal present at 5.3 ppm to 5.5 ppm, which belongs to the signal peak on the α-MSD end double bond (Within the red dashed box). This indicates that the PFS is obtained through chain transfer polymerization of α-MSD as the chain transfer agent and the polymer chain [28]. The ^1^H NMR spectrum of PCS exhibits similarities to that of PFS, except for the presence of a proton signal from the carboxyl group (–COOH) linked to the benzene ring at 13 ppm in Figure 1b. In addition, a strong signal peak originating from the double bond at the terminus of α-MSD is observed at around 5 ppm in Figure 1c,d.

**Figure 2:**
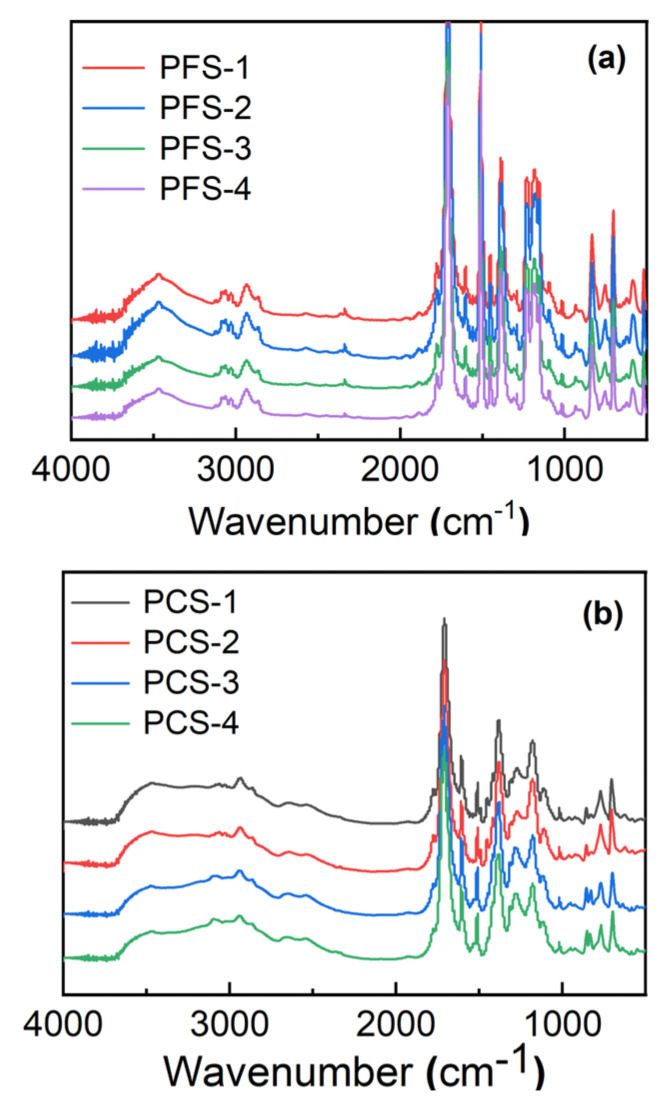
FTIR spectra recorded for (**a**) PFS and (**b**) PCS.

Figure 2a shows the FTIR spectrum of PFS. The absorption peaks are identified as follows: the stretching vibration of the amino group at 3104 cm^−1^, the ν_C=O_ absorption peak of benzene or olefin at 1716 cm^−1^, the ν_C=C_ skeletal vibrations of the benzene ring at 1601, 1584, and 1518 cm^−1^, the ν_C-N_ stretching vibration at 1393 cm^−1^, the ν_C=C_ bending vibrations of the aromatic ring at 1023 and 687 cm^−1^, and the ν_C-F_ stretching vibrations on the benzene ring at 1232 and 1151 cm^−1^. The -C-C- backbone peak of the macromolecular main chain is observed at 2923 cm^−1^. Figure 2b shows the FTIR spectrum of PCS. The absorption peaks include the ν_C=O_ absorption peak of benzene or olefin at 1712 cm^−1^, the ν_C=C_ skeletal vibrations of the benzene ring at 1604, 1579, and 1515 cm^−1^, the ν_C-N_ stretching vibration at 1395 cm^−1^, and the ν_C=C_ bending vibrations of the aromatic ring at 952 and 688 cm^−1^. These peaks confirm the successful synthesis of PFS and PCS.

**Figure 3:**
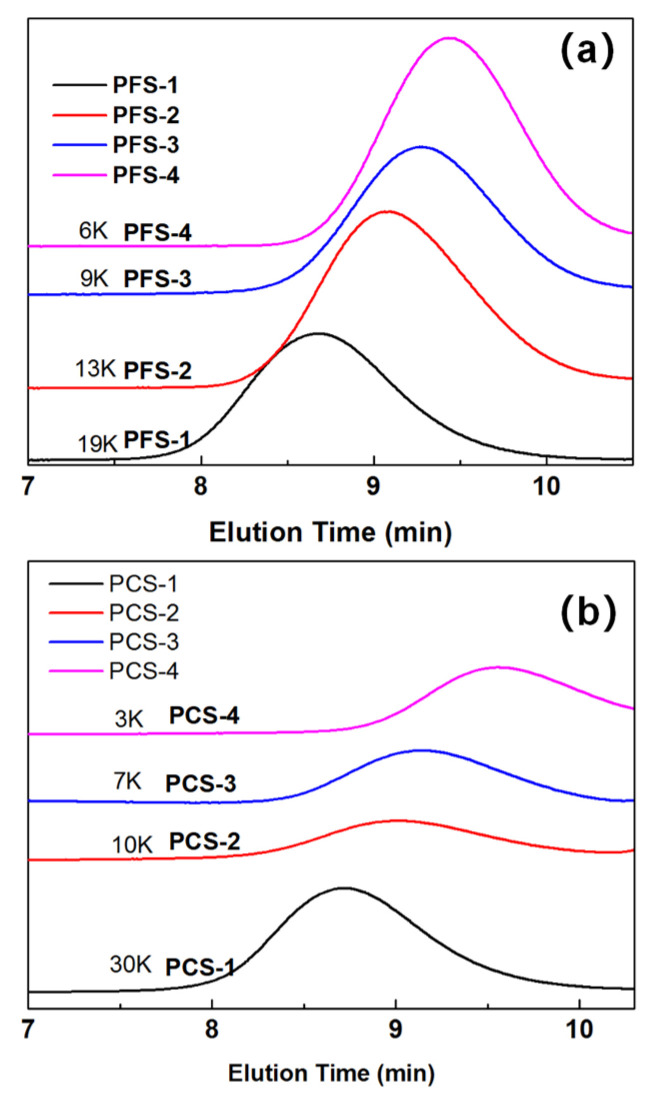
Tetrahydrofuran GPC curves obtained for a series of (**a**) PFS and (**b**) PCS.

The GPC curves of tetrahydrofuran-based PFS and PCS, obtained from Figure 3a,b, demonstrate a systematic decrease in molecular weight with increasing content of the chain transfer agent, α-MSD. The decrease in PCS molecular weight is particularly apparent, with a decrease of 20,000 kDa in molecular weight upon the addition of a small amount of α-MSD. These results suggest that α-MSD is an effective chain transfer agent for PFS and PCS and that molecular-weight-controlled maleimide-derivatized styrene copolymers can be prepared by controlling the amount of added α-MSD [48,49]. The similar distribution of GPC curves for PFS and PCS indicates excellent polymerization control in the free radical copolymerization process [50].

**Figure 4:**
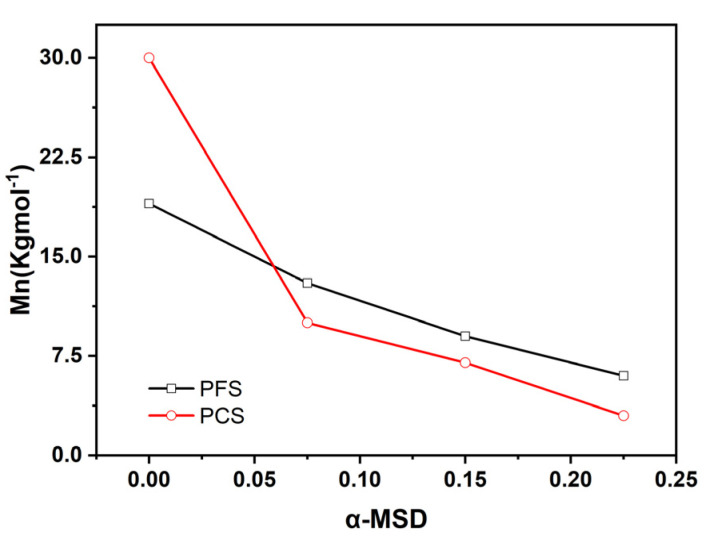
The number-average molecular weight of PFS and PCS in relation to the [α-MSD] curves at different masses.

As shown in Figure 4, there is an inverse correlation between the number-average molecular weight of PFS and PCS and the mass of [α-MSD]. The molecular weight of the polymers decreases with an increase in the mass of [α-MSD]. Initially, the molecular weight of PCS exhibits significant changes, followed by a similar trend as the molecular weight of PFS, both linearly decreasing with an increasing mass of [α-MSD]. This indicates that in this polymerization, the system with α-MSD as the CTA is a well-controlled polymerization system in terms of molecular weight [51,52].

**Figure 5:**
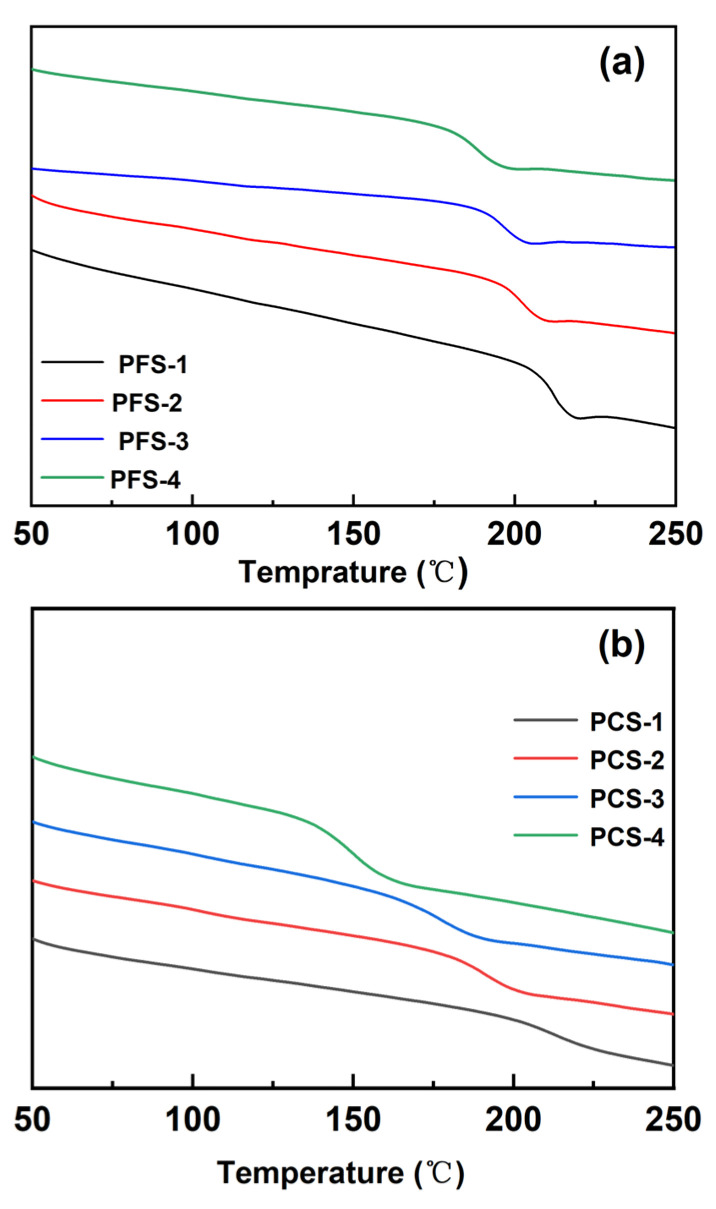
DSC curves obtained for a series of (**a**) PFS and (**b**) PCS.

A series of PFS and PCS with different molecular weights was prepared to study the thermal properties of these polymers upon addition of α-MSD to the free radical copolymerization system. The glass transition temperature (Tg) of the polymers was determined by DSC analysis. As shown in Figure 5, the Tg of both PFS and PCS decreases with a decrease in the molecular weight of the polymer chains, because due to the increased mobility of the chain end segments in shorter oligomeric chains, the chain end groups can provide more free volume [53,54]. With decreasing molecular weight, the proportion of chain end segments increases, leading to a lower Tg. The glass transition temperatures (Tg) of PFS-1, PFS-2, PFS-3, and PFS-4 were determined to be 215 °C, 192 °C, 176 °C, and 151 °C, respectively. Similarly, the Tg values of PCS-1, PCS-2, PCS-3, and PCS-4 were measured as 213 °C, 191 °C, 174 °C, and 149 °C, respectively. The results demonstrate that the glass transition temperatures of the PFS and PCS materials are closely comparable and exhibit a consistent trend with respect to molecular weight variation.

**Figure 6:**
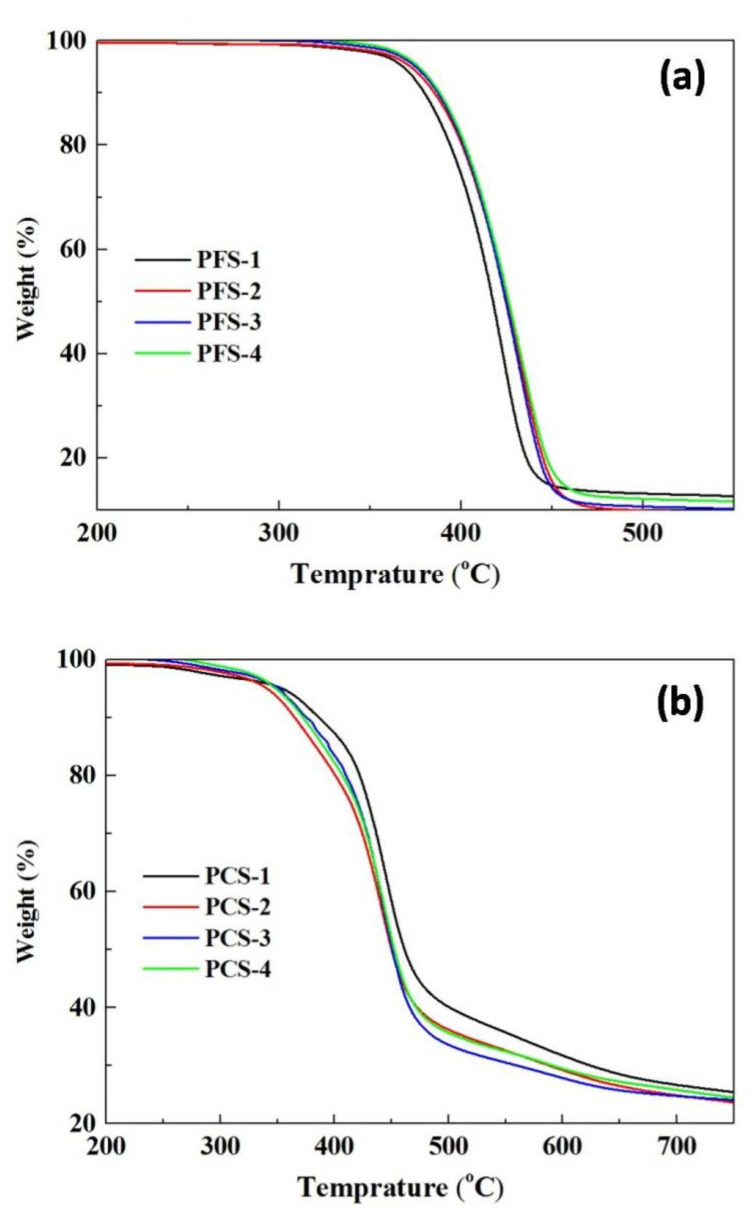
TG curves obtained for a series of (**a**) PFS and (**b**) PCS.

As shown in Figure 6, the influence of α-MSD on the PCS and PFS was studied using thermogravimetric analysis (TGA). After adding 0.075 g, 0.15 g, and 0.225 g of α-MSD to the reaction systems of PCS and PFS, a series of molecular weight reductions were observed, while the thermal stability exhibited only slight changes without significant overall variation. Both PCS and PFS displayed a single-step degradation process in the TG curves, and this pattern remained unchanged even after the addition of α-MSD. This indicates that the thermal stability of PCS and PFS is minimally affected by α-MSD, and PCS and PFS with different molecular weights can still maintain good thermal stability even with the incorporation of varying amounts of α-MSD.

**Figure 7:**
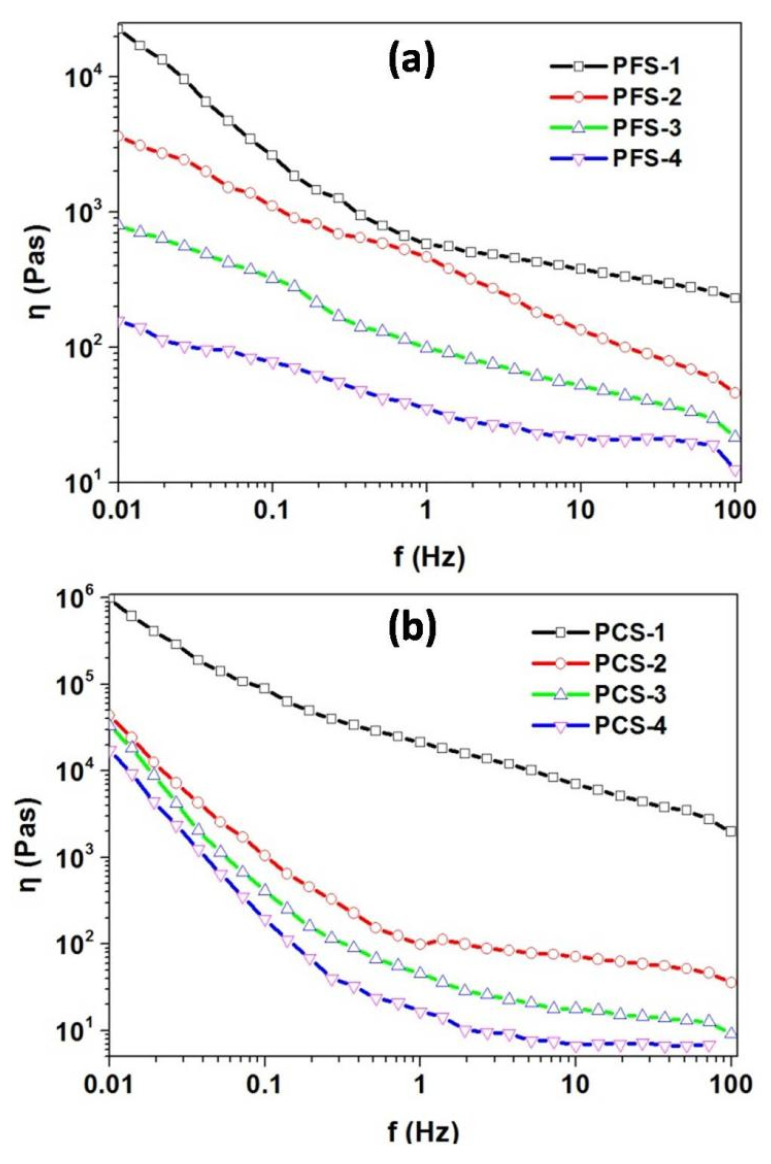
The dynamic viscosity curves obtained for a series of (**a**) PFS and (**b**) PCS.

The rheological behavior of polymers is an important indicator of their processability. Figure 7 demonstrates the variation in shear viscosity with shear rate for different molecular weight PFS and PCS. All PFS and PCS molecules exhibit shear-rate-dependent behavior, displaying shear-thinning behavior characteristic of pseudoplastic fluids, where the shear viscosity decreases with increasing shear rate [55,56,57]. This indicates that both PFS and PCS possess favorable processability. Additionally, both PFS and PCS display molecular-weight-dependent behavior, where the shear viscosity decreases with decreasing molecular weight. The molecular-weight-dependent behavior of PFS and PCS reveals the variation in polymer molecular structure. By incorporating α-MSD, the molecular weight of PFS and PCS can be controlled to further improve their processability.

### 2.2. Application as a Heat Resistance Agent

Recently, we reported the conventional free radical alternating copolymerization of styrene with N-phenylmaleimide (NPMI) and 4-CPMI, 4-FPMI in cyclohexanone [58,59,60]. Furthermore, the resulting copolymers PFS and PCS were utilized as thermal stabilizers in PA6. Previous research has demonstrated that PFS can induce a crystalline phase transition in PA6, promoting the formation of hydrogen bonds and enhancing the thermal resistance of PA6-based composites [60].

**Figure 8:**
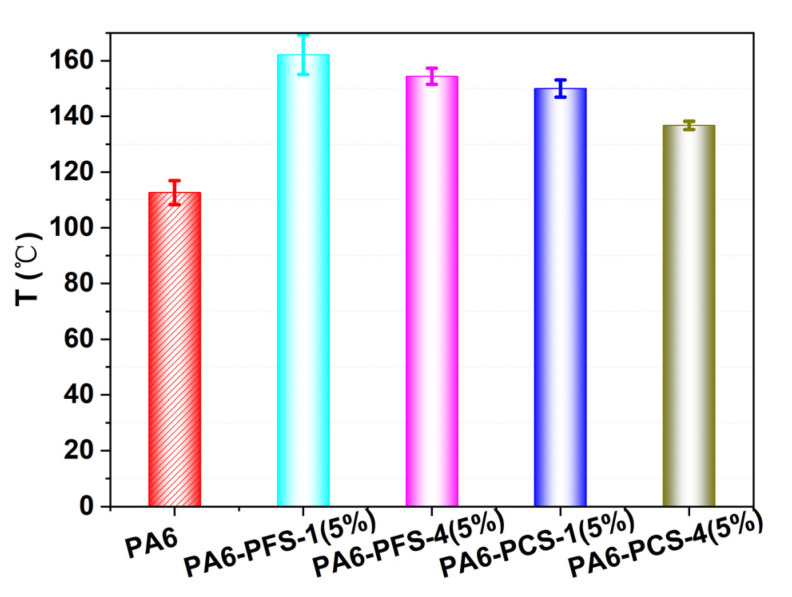
HDT obtained for a series of PA6, PA6-PFS and PA6-PCS.

HDT is commonly utilized as a physical property parameter to assess the ability of plastic materials to withstand short-term stress at elevated temperatures [61,62]. In the context of engineering plastics such as nylon, HDT holds particular significance, especially in applications within the automotive industry. The molecular weight of a polymer has an impact on the HDT. As the molecular weight of the polymer increases, the molecular chains tend to become entangled, leading to a decrease in chain mobility and an increase in HDT [63]. Therefore, a decrease in molecular weight would have a negative impact on HDT. However, in this study, the decrease in molecular weight of the heat-resistant agents PFS and PCS did not have a significant impact on the heat resistance of PA6 composites. As shown in Figure 8, PFS-4 and PCS-4 exhibited a significant decrease in molecular weight compared to PFS-1 and PCS-1. However, the HDT values of PA6-PFS-4 (5%) and PA6-PCS-4 (5%) only showed a slight decrease compared to PA6-PFS-1 (5%) and PA6-PCS-1 (5%). Moreover, the HDT values of PA6-PFS-4 (5%) and PA6-PCS-4 (5%) remained significantly higher than that of pure PA6. This indicates that low-molecular-weight PFS and PCS still exhibit a notable improvement in the heat resistance of PA6. Under the same conditions, PFS exhibited slightly better enhancement in the HDT value of PA6 than PCS, which can be attributed to the higher bond energy of the carbon–fluorine bonds and the strong hydrogen bonding between the PA6 matrix and PFS [64]. Previous XRD studies have indicated that the significant increase in HDT can be attributed to the strong hydrogen bonding interactions between the PA matrix and the PFS surface, which facilitates the formation of more perfect crystalline structures [60].

**Figure 9:**
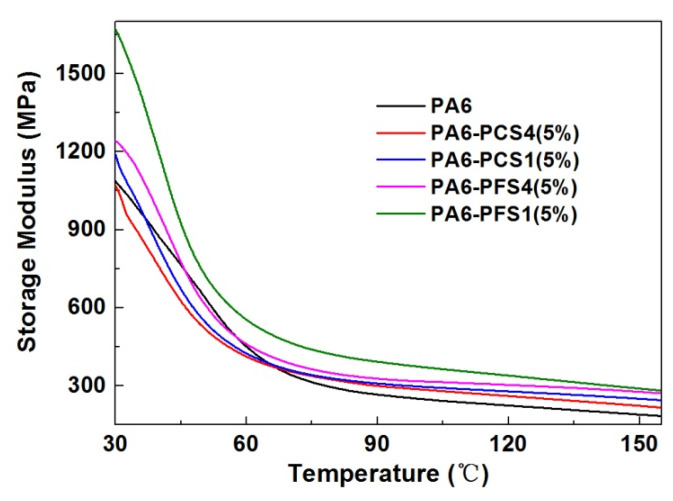
Storage modulus curves obtained for a series of PFS and PCS.

The relationship between temperature and the dynamic storage modulus of a series of PA6 composites was investigated using DMA. The storage modulus reflects the stiffness and load-bearing capacity of the material and is generally positively correlated with the rigidity of materials. As shown in Figure 9, the storage moduli of all samples exhibit a consistent trend with temperature variation, decreasing with increasing temperature. The storage modulus curve resembles an “L-shape” and can be divided into two regions. Within the temperature range of 30 °C to 65 °C, the storage modulus experiences a steep decline as the temperature rises. The increase in temperature triggers the motion of molecular chain segments, weakening intermolecular forces and transitioning the composite material from a glassy state to a rubbery state. In the temperature range of 65 °C to 150 °C corresponding to the rubbery region, the storage modulus tends to stabilize as the temperature rises. The storage moduli of PA6-PCS1 (5%) and PA6-PFS1 (5%) are higher than those of PA6-PCS4 (5%) and PA6-PFS4 (5%), which is consistent with the variation in HDT. This is attributed to the higher molecular weights of PCS1 and PFS1, requiring overcoming greater internal energy for molecular chain movement and thus exhibiting higher rigidity. The combination of PCS1 and PFS1 with PA6 effectively inhibits molecular chain migration, resulting in a composite material with increased stiffness. The storage modulus of the PA6-PFS series samples is higher than that of the PA6-PCS series samples. This indicates that the high bond energy of fluorine groups and their significant enhancement of molecular chain rigidity can more effectively increase the storage modulus of PA6.

## 3. Experimental Section

### 3.1. Materials

Maleic anhydride (MAH) was purchased from Tianjin damao chemical reagent factory (Tianjin, China) and used as received. Hydroquinone was purchased from Tianjin commie chemical reagent (Tianjin, China) Co., Ltd. and used as received. Styrene (St, CR) was purchased from Sinopharm chemical reagent (Shanghai, China) Co., Ltd. and was passed over an aluminum oxide column before usage to remove any inhibitor. 4-aminobenzoic acid and 4-fluoroaniline were purchased from Shanghai Aladdin biochemical technology (Shanghai, China) Co., Ltd. and used as received. Benzoyl peroxide (BPO) was recrystallized from methyl alcohol and stored in the fridge. (4-methylpent-1-ene-2,4-diyl) dibenzene (α-MSD) was purchased from TCI (Shanghai, China) Development Co., Ltd. Acetic anhydride and acetone were purchased from Chongqing sichuan east chemical (group) (Chongqing, China) Co., Ltd. and used as received. N, N-dimethyl formamide (DMF) was purchased from Taicang Hushi test reagent (Suzhou, China) Co., Ltd. and used as received. Cyclohexanone was purchased from Chengdu kelong chemical reagent plant (Chengdu, China) and used as received. Sodium acetate anhydrous was purchased from Chengdu kelong chemical reagent plant and used as received. Anhydrous sodium sulfate was purchased from Jiangsu powerful functional chemical (Jiangsu, China) Co., Ltd. and used as received. Nylon 6 (PA6, YH-800) was purchased from Yueyang petrochemical (Hunan, China) Co., Ltd. and used as received. N-p-fluorophenylmaleimide (4-FPMI) and N-p-carboxylphenylmaleimide (4-CPMI) were prepared as described in the literature [43,60].

### 3.2. Synthesis of P (N-p-fluorophenylmaleimide-alt-Styrene) (PFS)

As shown in Scheme 1 and Table 1, the free radical copolymerization of styrene with N-p-fluorophenylmaleimide (4-FPMI) was carried out in a 5 L mouth flask purged with dry nitrogen. The copolymerization process is as follows: 4-FPMI (191 g) and St (104 g) were added and dissolved in cyclohexanone (3 L) by stirring at 76 °C for 40 min before the initiator, BPO (7.5 g), was added. The reactor was heated by external circulating heated silicon oil, and the temperature was raised from 76 °C to 100 °C within half an hour. The copolymerization was then carried out at 100 °C for 4 h under a nitrogen atmosphere, followed by cooling to room temperature via an ice bath. The copolymer was then dissolved in acetone, and the solution was poured into an excess of methanol (precipitant) to precipitate the polymer, removing residual monomers and initiators. This procedure was repeated twice, and the final product was dried under vacuum at 100 °C for 48 h until constant weight was achieved.

### 3.3. Synthesis of P (N-p-carboxylphenylmaleimide-alt-Styrene) (PCS)

The free radical copolymerization of PCS was the same as that of PFS N-p-carboxylphenylmaleimide (208 g) and St (104 g).

### 3.4. Free Radical Chain Transfer Copolymerization of Styrene with 4-FPMI in Cyclohexanone

As shown in Scheme 1 and Table 1, the free radical chain transfer copolymerization of N-p-fluorophenylmaleimide (4-FPMI) with styrene was carried out in a 5 L mouth flask purged with dry nitrogen. The free radical chain transfer copolymerization process is as follows: 4-FPMI (191 g) and St (104 g) were added and dissolved in cyclohexanone (3 L) by stirring at 76 °C for 40 min before the chain transfer agent and initiator with different mass were added. The reactor was heated by external circulating heated silicon oil, and the temperature was raised from 76 °C to 100 °C within half an hour. The copolymerization was then carried out at 100 °C for 4 h under a nitrogen atmosphere, followed by cooling to room temperature via an ice bath. The copolymer was then dissolved in acetone, and the solution was poured into an excess of methanol (precipitant) to precipitate the polymer, removing residual monomers and initiators. This procedure was repeated twice, and the final product was dried under vacuum at 100 °C for 48 h until constant weight was achieved.

### 3.5. Free Radical Chain Transfer Copolymerization of Styrene with 4-CPMI in Cyclohexanone

Free radical chain transfer copolymerization of styrene with 4-CPMI was performed in cyclohexanone. The free radical chain transfer copolymerization of PCS was the same as that of PFS: N-p-carboxylphenylmaleimide (208 g) and styrene (St, 104 g) were used.

### 3.6. Preparation of PA6–Heat Resistance Agent Composites

After drying the heat resistance agent and PA6 in a vacuum at 100 °C for 12 h, they were blended, extruded, and pelletized in a miniature twin screw extruder. Then, injection molding was performed after drying. The extrusion temperature was divided into 10 parts in total, including nine sections from section 1 to section 9 and the die head. The temperatures were 190 °C, 200 °C, 210 °C, 215 °C, 220 °C, 225 °C, 230 °C, 235 °C, 240 °C, and 245 °C respectively.

### 3.7. Copolymer Characterization

^1^H NMR Spectroscopy. All 400 MHz ^1^H NMR spectra were recorded in *d*_6_-DMSO or CCl_3_D (concentration: 10 mg/mL) using a Bruker Ascend400 spectrometer JEOL Ltd. (Tokyo, Japan). The ^1^H spectra were referenced internally to the solvent peaks.

Fourier-transform infrared spectroscopy (FTIR). FTIR was performed using a Nexus-670 Fourier-transform infrared spectrometer (NICOLET Company, Waltham, MA, USA) in transmission mode with KBr pellets. The sample was mixed with KBr at a mass ratio of 1:100 in an agate mortar, ground into a fine powder, and pressed into a pellet. The scanning range was 400–4000 cm^−1^, with 32 scans and a resolution of 2 cm^−1^. The sample was scanned first, followed by background subtraction. The wavenumber was plotted on the x-axis, and absorbance was plotted on the y-axis.

Gel Permeation Chromatography (GPC). Analyses were performed using a Viscotek TDA302 Agilent Technologies (China) Co., Ltd. (Beijing, China) with the M GPC solvent/sample module in THF (1.0 mL/min, 30 °C). The concentration of the sample was 3 mg/mL, and the amount extracted each time was 1 μL. The sample was tested in four modes: RI, UV, RALS, and LALS. Calibration was achieved using polystyrene standards (M_w_ = 105 k).

Differential scanning calorimetry (DSC). The glass transition temperature (Tg) was measured using a TA Q10 (TA Instruments, Newark, DE, USA) system under a nitrogen atmosphere over a temperature range from 40 to 270 °C at a scan rate of 10 °C/min.

Thermogravimetic (TG). Measurements were performed on a TA Q50 (TA Instruments, Newark, DE, USA) system under nitrogen over a temperature range from 20 to 700 °C at a scan rate of 10 °C/min.

Dynamic viscosity (η). Measurements were performed on an advanced rheometric expansion system (ARES, TA Instrument, New Castle, DE, USA) using the parallel-plates mode. The rheological measurement dimensions were 10 mm × 10 mm, and the gap was 1 mm. The measurements were conducted at 230 °C. The samples were loaded onto the bottom plate of the instrument and heated until fully melted. Frequency sweeps were conducted at 230 °C in the frequency range of 0.01 rad/s to 500 rad/s within the linear region.

Heat deflection temperature (HDT). Measurements were performed on a thermal deformation temperature tester (Eden Prairie, MN, USA. MTS ZWK1000). The measurements were carried out according to GB/T1634.1-2004; Determination of Vicat softening temperature of plastics—Part 1: General test method. China Standards Press: Beijing, China, 2004. under a load of 0.45 MPa and a temperature rate of 120 °C/h.

Dynamic Mechanical Analysis (DMA). Measurements were carried out on a Q800 (TA Instruments, Newark, DE, USA) DMA. The specimen dimensions were 60 mm in length, 10 mm in width, and 4 mm in thickness. The measurement frequency was 1 Hz, and the heating rate was 2 °C/min. The temperature range was from 25 °C to 120 °C. High-temperature measurements were conducted under a dry N_2_ flow.

## 4. Conclusions

By using 4-methylpent-1-ene-2,4-diyl) diphenyl (α-MSD) as a chain transfer agent, the successful preparation of the synthesis of P (N-p-carboxylphenylmaleimide-alt-Styrene) (PCS) and synthesis of P (N-p-fluorophenylmaleimide-alt-Styrene) (PFS) with different molecular weights was achieved through free radical copolymerization. Different molecular weight PCS and PFS were then compounded with PA6 to prepare a series of PA6 composite materials. The effect of α-MSD as a chain transfer agent on the molecular regulation of PCS and PFS, as well as the influence on thermal properties, rheological behavior, and the impact of introducing different molecular weight PCS and PFS on PA6 HDT and the dynamic storage modulus were investigated. The GPC curve analysis demonstrated that α-MSD as a chain transfer agent can effectively regulate the molecular weight of PCS and PFS. The DSC analysis showed that the reduction in molecular weight would slightly lower the Tg of PCS and PFS, with a more significant effect observed in PCS. The TGA analysis indicated that the reduction in molecular weight had little effect on the thermal stability of PCS and PFS. The dynamic curve analysis demonstrated that decreasing the molecular weight effectively reduced the viscosity of PCS and PFS, which would benefit their processing performance. The HDT analysis and DMA confirmed that the introduction of PCS and PFS could effectively enhance the HDT and stiffness of PA6, but the impact of molecular weight reduction led to a slight decrease in HDT and stiffness.

## Figures and Tables

**Table 1:** Copolymerization of styrene with 4-FPMI (4-CPMI) in cyclohexanone.

Entry No.	Time (h)	Temperature (°C)	BPO (g)	α-MSD (g)	4-FPMI (g)	4-CPMI (g)	St (g)
PFS-1	4	100	0.075	0	1.91		1.04
PFS-2	4	100	0.075	0.075	1.91		1.04
PFS-3	4	100	0.075	0.15	1.91		1.04
PFS-4	4	100	0.075	0.225	1.91		1.04
PCS-1	4	100	0.075	0		2.17	1.04
PCS-2	4	100	0.075	0.075		2.17	1.04
PCS-3	4	100	0.075	0.15		2.17	1.04
PCS-4	4	100	0.075	0.225		2.17	1.04

## Data Availability

The original contributions presented in this study are included in the article. Further inquiries can be directed to the corresponding authors.
